# Molecular pathway profiling of T lymphocyte signal transduction pathways; Th1 and Th2 genomic fingerprints are defined by TCR and CD28-mediated signaling

**DOI:** 10.1186/1471-2172-13-12

**Published:** 2012-03-14

**Authors:** Ruben L Smeets, Wilco WM Fleuren, Xuehui He, Paul M Vink, Frank Wijnands, Monika Gorecka, Henri Klop, Sussane Bauerschmidt, Anja Garritsen, Hans JPM Koenen, Irma Joosten, Annemieke MH Boots, Wynand Alkema

**Affiliations:** 1 Department of Immune Therapeutics, Merck Research Laboratories (MRL), MSD, Oss, the Netherlands; 2 Computational Drug Discovery (CDD), CMBI, NCMLS, Radboud University Nijmegen Medical Centre, Nijmegen, The Netherlands; 3 Netherlands Bioinformatics Centre (NBIC), Nijmegen, The Netherlands; 4 Department of Molecular Design and Informatics, Merck Research Laboratories (MRL), MSD, Oss, the Netherlands; 5 NIZO Food Research, Ede, The Netherlands; 6 Department of Laboratory Medicine, laboratory for Clinical Chemistry, Radboud University Medical Centre, Nijmegen, The Netherlands; 7 Department of Laboratory Medicine, laboratory for Medical Immunology, Radboud University Medical Centre, Nijmegen, The Netherlands; 8 Department of Rheumatology and Clinical Immunology, University Medical Centre Groningen, University of Groningen, Groningen, The Netherlands; 9 Department of Laboratory Medicine, laboratory for Clinical Chemistry, Radboud University Medical Centre, Nijmegen, Geert Grooteplein 10, Postbus 9101, 6500 HB Nijmegen, The Netherlands

**Keywords:** Signal transduction pathways, Gene expression profiling, T lymphocytes, Th1 and Th2 development

## Abstract

**Background:**

T lymphocytes are orchestrators of adaptive immunity. Naïve T cells may differentiate into Th1, Th2, Th17 or iTreg phenotypes, depending on environmental co-stimulatory signals. To identify genes and pathways involved in differentiation of Jurkat T cells towards Th1 and Th2 subtypes we performed comprehensive transcriptome analyses of Jurkat T cells stimulated with various stimuli and pathway inhibitors. Results from these experiments were validated in a human experimental setting using whole blood and purified CD4+ Tcells.

**Results:**

Calcium-dependent activation of T cells using CD3/CD28 and PMA/CD3 stimulation induced a Th1 expression profile reflected by increased expression of T-bet, RUNX3, IL-2, and IFNγ, whereas calcium-independent activation via PMA/CD28 induced a Th2 expression profile which included GATA3, RXRA, CCL1 and Itk. Knock down with siRNA and gene expression profiling in the presence of selective kinase inhibitors showed that proximal kinases Lck and PKCθ are crucial signaling hubs during T helper cell activation, revealing a clear role for Lck in Th1 development and for PKCθ in both Th1 and Th2 development. Medial signaling via MAPkinases appeared to be less important in these pathways, since specific inhibitors of these kinases displayed a minor effect on gene expression. Translation towards a primary, whole blood setting and purified human CD4+ T cells revealed that PMA/CD3 stimulation induced a more pronounced Th1 specific, Lck and PKCθ dependent IFNγ production, whereas PMA/CD28 induced Th2 specific IL-5 and IL-13 production, independent of Lck activation. PMA/CD3-mediated skewing towards a Th1 phenotype was also reflected in mRNA expression of the master transcription factor Tbet, whereas PMA/CD28-mediated stimulation enhanced GATA3 mRNA expression in primary human CD4+ Tcells.

**Conclusions:**

This study identifies stimulatory pathways and gene expression profiles for in vitro skewing of T helper cell activation. PMA/CD3 stimulation enhances a Th1-like response in an Lck and PKCθ dependent fashion, whereas PMA/CD28 stimulation results in a Th2-like phenotype independent of the proximal TCR-tyrosine kinase Lck. This approach offers a robust and fast translational in vitro system for skewed T helper cell responses in Jurkat T cells, primary human CD4+ Tcells and in a more complex matrix such as human whole blood.

## Background

Activation of T helper 0 (Th0) cells leads to differentiation into several lineages. These lineages include the Th1 and Th2 subsets as well as the more recently described subsets such as induced T regulatory cells and Th17 cells. The Th1 cells protect against intracellular pathogens and are in general characterized by their ability to produce IFNγ, IL-2 and TNFα and express the Th1-specific transcription factor T-bet. The Th2 subset, which is involved in the defense against extracellular pathogens, is characterized by the production of IL-4, IL-5 and IL-13 and is controlled by the master transcription factor GATA3 [1,2].

In a proper functioning immune system, these different T helper subsets are well-balanced and co-operate to eliminate invading pathogens and to maintain homeostasis. Hyper activation of one T helper subset, however, can tip the balance from health towards disease, in which Th2-overshoot can lead to inappropriate immune responses leading to diseases like allergy and asthma. Alternatively, overshoot towards a Th1 or Th17-phenotype can cause autoimmune diseases, like rheumatoid arthritis and multiple sclerosis [3,4].

For effective CD4 T cell activation, the antigen-presenting cell (APC) provides a key contact point to facilitate T cell activation and polarization towards different T helper subsets. A crucial event in this process is the interaction between the antigen presented via the MHCII receptor and the TCR receptor (signal 1). The nature of activation, defined by the strength of the TCR stimulation, can affect T helper cell polarization towards Th1 or Th2, in which a high affinity interaction favors Th1 development and low affinity drives Th2 development [5-8]. Besides the TCR signal transduction, an additional signal is provided by the APC in the form of a co-stimulatory signal (signal 2). This signal is provided via CD28-B7 interaction and has been shown to be important for effective T cell activation [9]. Furthermore, CD28-mediated co-stimulation has been implicated in effective polarization of T cells towards a Th2 phenotype [10,11]. Also other co-stimulatory molecules, including ICOS and OX40, have been positively correlated with Th2 differentiation [12,13]. The results from these studies underline the importance of both signal 1 and signal 2, but also underline the complexity of these integrated signaling pathways.

The cascade of biochemical events, linking cell surface receptor engagement to cellular responses has been a focus of many studies. Detailed investigation of these signal transduction events has led to identification and functional characterization of many kinases and phosphatases downstream of the TCR and CD28-receptor. TCR ligation results in the recruitment of p56Lck (Lck), a proximal TCR Src family kinase, which kick-starts the signal transduction cascade leading to phosphorylation of the ITAM motifs in the TCR, which recruits and activates ZAP70 [14]. This initial step leads to the activation of PLCγ that hydrolyzes PIP_2 _into IP_3_, which is the second messenger molecule responsible for the sustained intracellular calcium flux in T cells. CD28-ligation on T cells results in the recruitment of PI_3_K, with PIP_2 _and PIP_3_, which serve as pleckstrin homology (PH) domain membrane anchors. Via this mechanism PDK1 and PKB/Akt are recruited and regulate several pathways that increase cellular metabolism [15]. Additionally, CD28-signaling has been shown to initiate NFκB signaling, via a mechanism that is functionally linked through recruitment of PKCθ to CD28 in the immunological synapse [16-18].

Members of the Mitogen-activated protein kinase family, which can be activated via TCR signaling, also play a role in the differentiation of Th1 and Th2 subsets. In a thorough review by Dong et al., the role of p38, JNK and ERK in T helper cell differentiation has been outlined [19]. ERK is important for Th2 differentiation, whereas p38 and JNK2 appear to be involved in Th1 development.

TCR/CD3 stimulation and CD28 stimulation alone are weak activators of T cell signaling. It is generally conceived that CD28 signaling merely acts as a signal potentiator on top of the initiator signal mediated via the TCR/CD3. Ledbetter and June et al. described that CD28 stimulation in the absence of cross-linking on top of PMA stimulation can activate T cells, without increasing calcium flux [20,21]. This suggests that co-stimulatory pathways synergize with biochemical pathways induced via the TCR. Whether CD28 ligation, in the absence of TCR signaling, leads to activation and differentiation has not been fully explored.

These findings show that effective T cell activation and differentiation towards effector subsets is the result of precise integration of multiple signaling routes. To explore the pathways underlying these distinct routes towards T cell activation and differentiation we used comprehensive biochemical characterization and gene expression profiling of Jurkat T cells that were activated with various co-stimulatory signals in the presence of various inhibitors of specific signaling routes.

With this approach we identified specific PMA/CD3 and PMA/CD28 signal transduction and genomic fingerprints. PMA/CD3 stimulation induced a Th1 phenotype, dependent on both Lck and PKCθ, whereas PMA/CD28 stimulation, which is independent of TCR-mediated activation of Lck, resulted in a profound activation of T cells, skewing towards a Th2 phenotype.

## Results and discussion

### Activation of Jurkat T cells by various stimuli leads to differential signaling fingerprints

**Figure 1:**
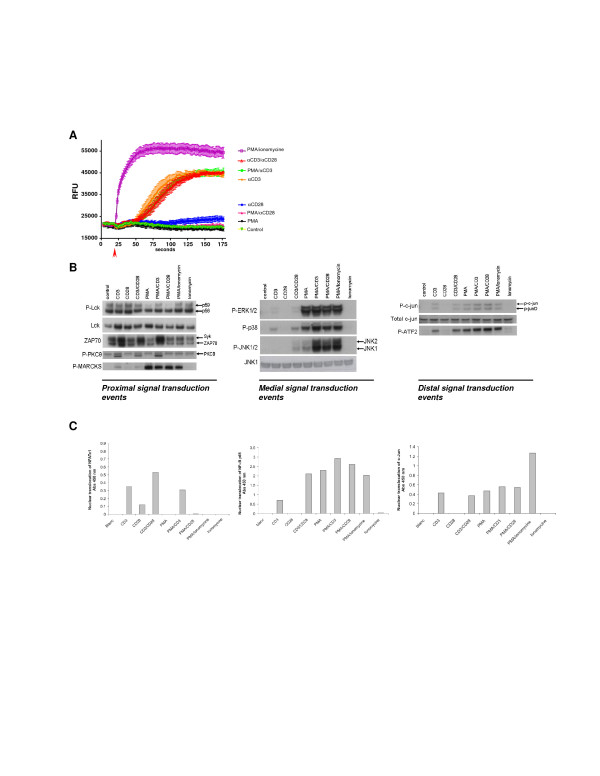
**Signal transduction in Jurkat T cells**. Jurkat T cells were stimulated with different combinations of stimuli in order to elucidate the different signal transduction pathways. **A**; Jurkat T cells were stimulated as indicated and intracellular Ca^2+ ^release was monitored over/in time. **B**; Intracellular signal transduction routes were charted via phosphoanalysis using western blot. Jurkat T cells were stimulated for 15 min using different stimulations. Proximal (Lck, ZAP70, PKCθ and the PKC substrate MARCKs), medial (MAPK phosphorylation) and distal (c-Jun and ATF2) signaling was monitored based on the phosphorylation status of the described proteins. **C**; Nuclear translocation of the transcription factors NFAT, NFkB and c-Jun was evaluated 15 minutes after stimulation.

Jurkat T cells were activated by anti-CD3, anti-CD28, PMA, or ionomycin or combinations of these single stimuli, in order to map the contribution of these stimuli towards the activation of proximal, medial and distal signal transduction pathways. As shown in Figure 1A, CD3-stimulation and ionomycin/PMA were able to increase intracellular levels of Ca^2+^. Interestingly, neither CD28 nor PMA stimulation alone, affected intracellular Ca^2+ ^levels. As expected, CD3-signaling resulted in an Lck-dependent phosphorylation of ZAP70 (Figure 1B). Stimuli containing PMA directly activated the MAPK pathway, which is reflected by the phosphorylation of ERK, P38 and JNK (Figure 1B). Furthermore, PMA addition directly activated PKC which was not reflected in the autophosphorylation of PKCθ, but was clearly detectable on the phosphorylation of the PKC substrate MARCKS (Figure 1B). CD3-mediated stimulations and PMA-induced stimulations resulted both in the activation of AP1 family transcription factors c-Jun and ATF2 (Figure 1B). Analysis of nuclear translocation of NFATc1 and c-Jun (AP1)/NFκB p65, as part of the distal signaling events revealed that indeed CD3-mediated signaling induced both NFAT and c-Jun/NFκB, of which the latter pathways were potentiated by CD28-mediated signaling (Figure 1C). In line with the calcium release from the ER, PMA or PMA/CD28-mediated signaling did not induce NFAT nuclear translocation but highly activated the CD28 responsive element transcription factors c-Jun and NFκB p65 (Figure 1C). These results indicate that two distinct co-stimulatory profiles can be identified. A CD3/28 and PMA/CD3 stimulus that signals via Lck, increasing intracellular Ca^2+ ^and activating NFAT, and a PMA/CD28 calcium independent (co)-stimulatory activation signaling via PKCθ and MARCKS. Next, the molecular mechanisms involved in these signaling pathways were further explored in genomics studies.

### Differential regulation of genes after PMA/CD3 and CD3/28 Vs PMA/CD28 stimulation

**Figure 2:**
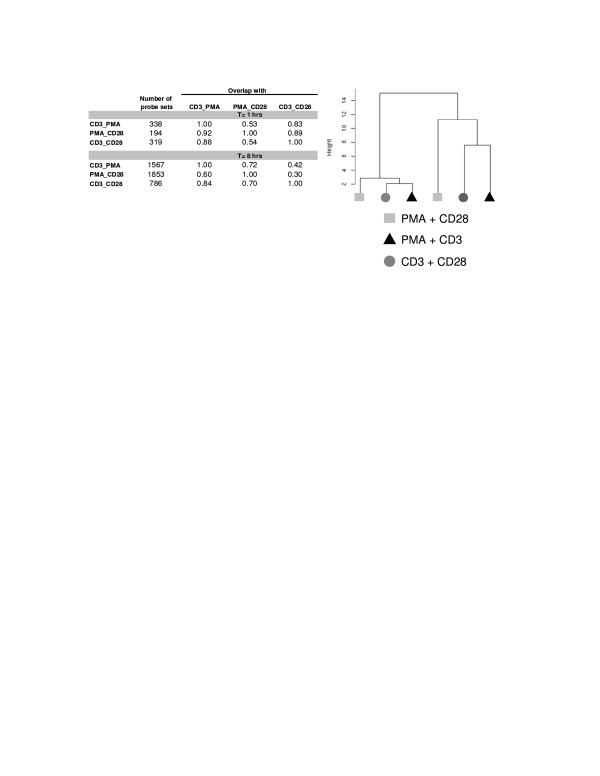
**PMA/CD3 and CD3/28 stimulations differ from PMA/CD28 stimulation**. The table shows the number of regulated probe sets and their overlap at 1 and 8 hrs following stimulation with CD3/PMA, PMA/CD28 and CD3/CD28 (using a fold change cut off of 2 and a p-value cut off of < 1.10^-6^). The right panel shows a hierarchical clustering of the data using the data for the regulated probe sets. Most of the variation is caused by the time difference (1 hr vs 8 hrs). At both time points the PMA/CD28 stimulus was clearly different from the CD3/CD28 and CD3/PMA stimulus. Repeating this analysis with different values for the fold change and p-value cut off yielded essentially the same results for the multivariate analysis.

In order to further characterize the different signal transduction events induced by different (co)-stimulatory signals, we performed a first gene expression experiment with Jurkat T cells that were stimulated for 1 or 8 hours with PMA/CD3, CD3/28 and PMA/CD28. It appeared that after 1 hour a limited response on transcription level was seen, whereas after 8 hours of stimulation, several hundreds of genes were regulated. Furthermore, PMA/CD3 and PMA/CD28 regulate more genes compared to CD3/28, reflecting the strength of the stimuli used (Figure 2). Multivariate analysis by principal component analysis and hierarchical clustering showed that the 3 stimuli lead to clearly distinct gene expression profiles. At both time points the profile induced by PMA/CD28 is clearly distinct from the profiles induced by PMA/CD3 and CD3/28 (Figure 2).

### Differentially regulated genes CCL1 and IL-2 are profile-specific secreted proteins

**Figure 3:**
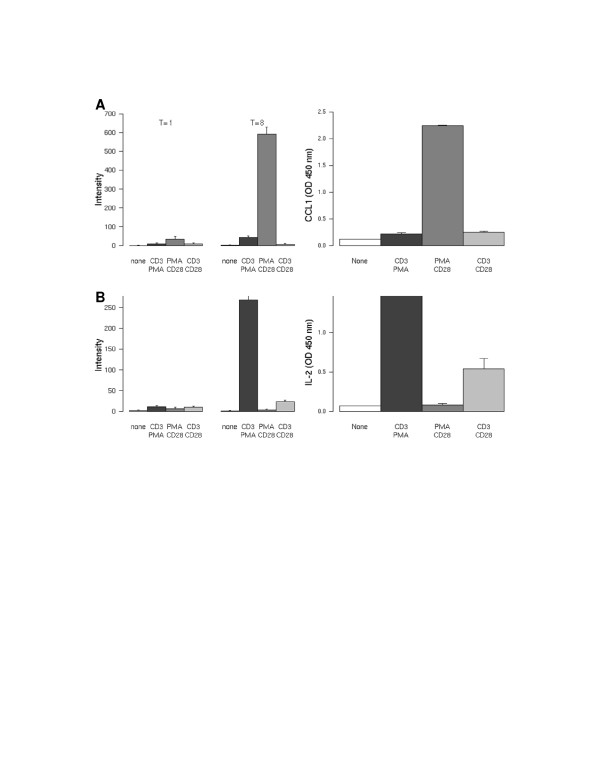
**Differential regulation of IL-2 and CCL1**. Jurkat T cells were activated for 1 and 8 hours using different stimuli (as indicated). Differentially regulated genes of which the proteins were secreted were identified and the highest ranking genes were selected. **A**; regulation of the CCL1 mRNA is highly and differentially regulated after PMA/CD28 stimulation. Right panel shows the validation of the mRNA levels on protein level, secreted by activated Jurkat T cells. **B**; regulation of IL-2 mRNA is highly and differentially regulated after CD3/28 and PMA/CD3 stimulation. Right panel shows the validation of the mRNA levels on protein, secreted by activated Jurkat T cells.

Gene profiles of the differential stimuli were ranked on the level of induction and evaluated on whether or not the translated protein is secreted. This resulted in the identification of the PMA/CD28-specific transcript CCL1 (Figure 3A), the CD3-specific transcripts IL-2 (Figure 3B) and XCL1/2 (data not shown). Small but significant inductions of these genes were observed after 1 hour of stimulation for both CCL1 and IL-2. However, both genes were highly induced after 8 hours of stimulation. The secretion of the protein 24 hours after stimulation reveals an identical profile compared to the mRNA (Figure 3A/B, right hand panel).

### Pathway profiling with multiple stimuli and inhibitors

To investigate the contribution of proximal, medial and distal signaling events on the CD3/28, PMA/CD3 and PMA/CD28 stimuli, we performed a second gene expression profiling experiment with different selective inhibitors, including proximal kinase inhibitors Lck (A420983), PKCθ (AEB071), medial MAPK inhibitors PD98059 (MEK/ERK), SP600125 (pan JNK), Org 48762-0 (P38) and the distal Calcineurin (Cn) inhibitor Cyclosporin A (CsA). Jurkat T cells were stimulated with PMA, CD28 and CD3 alone and combinations thereof in the presence of the above mentioned inhibitors. Based on the results of the first gene expression profiling experiment, we chose evaluated gene expression after 8 hours of stimulation. A principal component analysis on the ratio data set is shown in Additional file 1: Figure S1. It appeared that the PMA/CD28, CD3/CD28 and PMA/CD3 co-stimuli induced several hundreds of genes, whereas the effect of a single stimulus was smaller, with the exception of the PMA single stimulus (Additional file 1: Figure S1, Additional file 2: Table S1). The gene set induced by a PMA stimulus showed a larger overlap with the genes induced by the PMA/CD28 stimulus than with the CD3/PMA induced gene set. Whereas PMA and CD3 as a single stimulus induce a large number of genes, CD28 elicits only a minor effect. It can be observed that CsA, AEB071 and A420983 induce the largest effects on gene regulation, whereas the inhibitors of the MAPK pathway only have a minor effect on gene expression. This finding is corroborated by the number of regulated genes, showing that the MAPK inhibitors only regulate a small number of genes whereas A420983, CsA and AEB071 regulate many genes (Additional file 2: Table S1). A420983 and CsA only show a significant effect on the PMA/CD3 and CD3/CD28 pathways, in which the effect of CsA is smaller than the effect of A420983. AEB071 is the only compound that shows also a significant effect on the PMA/CD28 induced pathway. These analyses were rerun with different settings for the thresholds used for gene selection. In all cases similar results were obtained indicating that the results were not critical dependent on the thresholds settings that were used.

**Figure 4:**
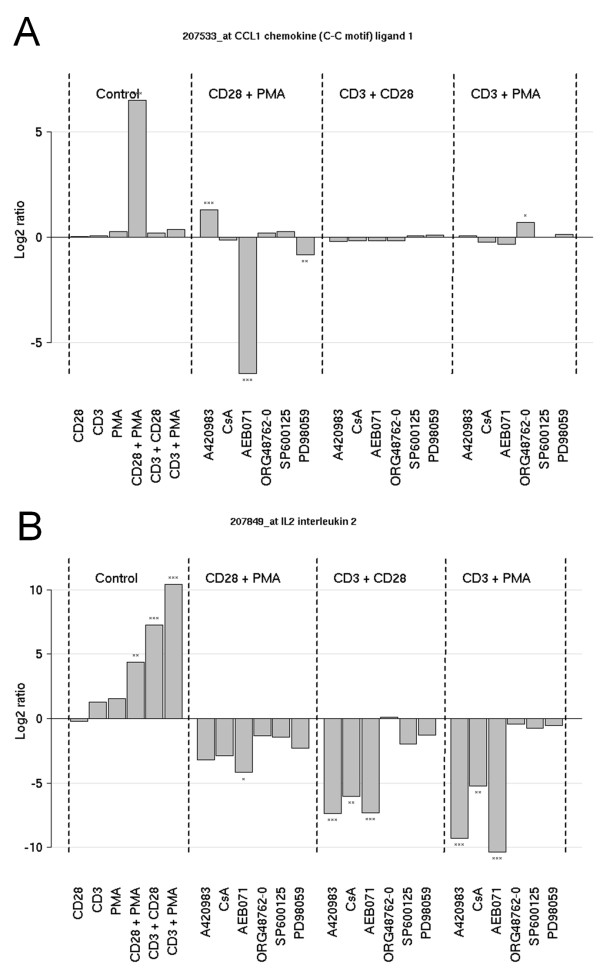
**Involvement of signal transduction pathways on differentially regulated genes**. Jurkat T cells were stimulated as indicated previously and the involvement of different signaling pathways on gene regulation was elucidated using inhibitors of specific pathways, including Lck (1 μM), PKC (10 μM), Calcineurin (1 μM), and MAPKs p38, JNK, and MEK/ERK (all 10 μM). **A**; Regulation of CCL1 mRNA in Jurkat T cells after stimulation with PMA/CD28-, CD3/28- or PMA/CD3-and co-incubated and cultured for 8 hours in the presence or absence of signal transduction pathway inhibitors. **B**; Regulation of IL-2 mRNA in Jurkat T cells after stimulation with PMA/CD28-, CD3/28- and PMA/CD3- and co-incubated and cultured for 8 hours in the presence or absence of signal transduction pathway inhibitors. The height of the bars represent the average intensity of 3 biological replicates, the error bars indicate the variation (min and max values). Significance of changes in expression level between the stimulated sample and the sample that was stimulated in the presence of the pathway inhibitor is indicated as follows; *** *P *value < 0.0001; ** 0.01 <*P *value > 0.001* 0.05 <*P *value > 0.01.

Inspection of the profiles of CCL1 and IL-2 revealed that CCL1 mRNA is highly induced via the PMA/CD28 pathway. This induction is depending on PKC signaling and negatively regulated via Lck signaling. Apparently this effect was upstream of Cn, since the inhibitor CsA did not increase CCL1 mRNA induction (Figure 4A).

**Figure 5:**
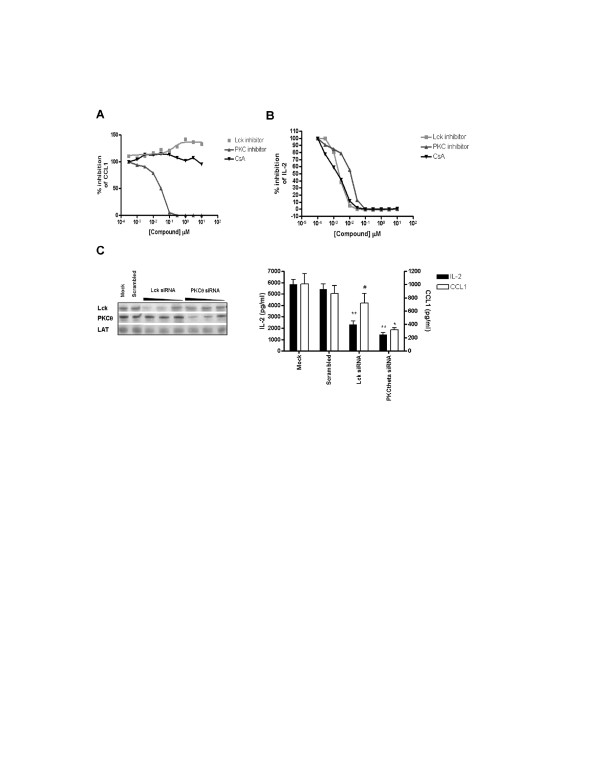
**PMA/CD28-induced CCL1 production is not dependent on the Lck/Cn pathway**. Jurkat T cells were stimulated using PMA/CD3 and PMA/CD28 in the presence of Lck (A420983), Cn (CsA) and PKC (AEB071) pathway inhibitors for 24 hours. Dose- response effects of the inhibitors were evaluated on the production of CCL1, after PMA/CD28 stimulation (**A**) and IL-2 after PMA/CD3 stimulation (**B**) in supernatant of the cell cultures. The data are representative for 3 independent experiments. **C**; Knock down of Lck and PKCθ resulted in a clear dose-dependent reduction of the protein (500, 100, 20 nM siRNA). 24 hour culture supernatants were collected after stimulation with PMA/CD28 and PMA/CD3 and the effect of knock down on respectively CCL1 and IL-2 was determined. Data of two independent experiments are presented as mean + SEM. Significance of differences are indicated by ** *p *< 0.01, *p < 0.05, # no difference using a one-way ANOVA with a Bonferroni's post-hoc test. N.b the values shown in this figure are log2 values no to be confused with the intensity values as shown in figure 3.

As expected, PMA/CD3 and CD3/28 stimuli and to a lesser extent PMA/CD28 resulted in a marked expression of IL-2, which is highly depending on the Lck/Cn signal transduction pathway, and the PKC pathway. Interestingly inhibition of MAPK signaling (with the exception of the MEK/ERK pathway) does not affect IL-2 mRNA induction (Figure 4B). These effects on CCL1 and IL-2 production by inhibitors of the Lck/Cn and PKC pathway were further substantiated in a full dose-response experiment. Figure 5A shows that indeed AEB071 dose-dependently inhibited PMA/CD28-induced CCL1 production, which is slightly enhanced in the presence of an Lck inhibitor and which is not affected by Cn inhibition via CsA. IL-2 production can be blocked by inhibition of both the Lck/Cn and PKC pathway (Figure 5B). The involvement of Lck in the CD3-mediated pathway and PKC in the CD3 and CD28-mediated pathways was further confirmed by the knock-down of both kinases under the distinct stimuli. Knock-down of Lck did not affect PMA/CD28-induced CCL1 production, whereas knock-down of PKCθ resulted in significant inhibition of both IL-2 and CCL1 (Figure 5C). These results clearly show that PMA/CD28-induced gene profiles are highly depending on PKCθ signaling pathways but are independent of Lck/Cn and MAPK signaling pathways, whereas CD3-mediated signaling pathways are dependent on both Lck/Cn and PKCθ signal transduction and independent on MAPK signaling events.

### PMA/CD3, PMA/CD28 and CD3/CD28 induce distinct genomic fingerprints

**Figure 6:**
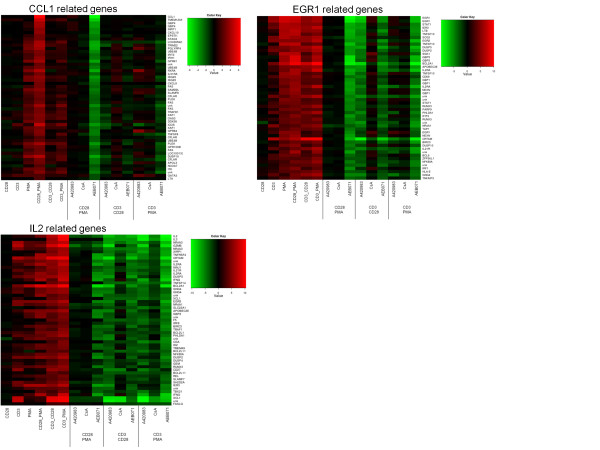
**Heatmap of stimulation dependent gene clusters**. Top left panel: CCL1 cluster characterized by induction of genes exclusively following CD28/PMA stimulation and subsequent repression by AEB071. Bottom left panel: IL-2 cluster characterized by induction of genes by all stimulations, and down-regulated by A420983 and AEB071 and to a lesser extent by CsA. Top right panel: EGR1 cluster characterized by induction of genes by all stimulations, but down-regulated only by AEB071 when stimulated with PMA/CD28 or PMA/CD3. A red color denotes an up-regulation, a green color a down-regulation. The MAPK inhibitors induced very little regulation and have been omitted from this figure for clarity. The gene lists shown in this figure, with extended annotation and their distance to the CCL1, IL-2 and EGR1 profiles are provided in Additional files: File S1, S2 and S3.

The above analysis indicated that treating Jurkat T-cells with multiple combinations of stimuli and inhibitors highlights pathways that are regulated by specific combinations of stimulus and inhibitor, revealing the involvement of certain kinases as signaling hub under specific stimulatory conditions. In order to identify additional genes in the pathways that are exemplified by CCL1 and IL-2, we searched for genes with similar profiles to these pathway genes. Figure 6 shows genes related to CCL1, identified by a strong up regulation following PMA/CD28 stimulation only and a down regulation by AEB071. Interestingly, besides CCL1, which is a chemo attractant for Th2 cells, many Th2-associated genes co-clustered with CCL1, including GATA3, Itk, RXRA, c-FLIP (CFLAR), ICOS and the IL-31 receptor and also other genes that are associated with Th2 development (See Figure 6; CCL1 gene cluster).

Likewise a very specific IL-2 profile can be constructed by selecting genes that are up regulated under all three conditions and down regulated by all three inhibitors, and by which CsA is the weakest inhibitor (see Figure 6; IL-2 cluster). In this gene cluster appeared to be Th1-associated genes, including the Th1 master transcription factor Tbet (TBX21), Th1 chemokine XCL1/2, IFNγ, granzyme, RUNX3, FASL, OX40L (TNFRSF4), CD27, and the IL-21 receptor. Of note, inhibition of both Lck and Cn under PMA/CD3 stimulation enhanced the expression of Th2 master transcription factors GATA3 and RXRA, but also peptidoglycan recognition protein 4 (PGLYRP4) and G protein-coupled receptor 84 (GPR84).

**Figure 7:**
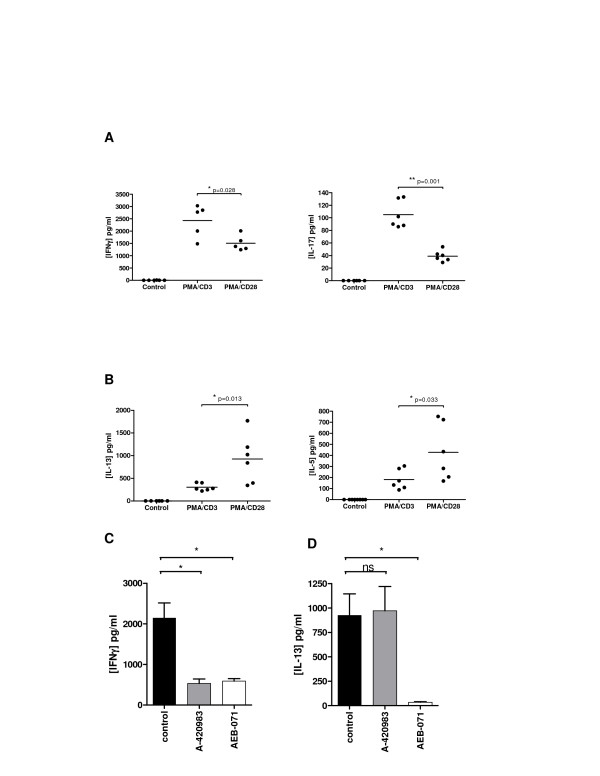
**PMA/CD3 and PMA/CD28 stimulation differentially modulate primary T cell cytokine responses in human healthy donor blood**. Healthy donor blood was stimulated with PMA/CD3 and PMA/CD28 and cultured for 24 hours in the presence or absence of pathway inhibitors for Lck and PKC. Culture supernatants were analyzed on **A**; IFNγ, IL-17 (Th1/17 cytokines) and **B**; IL-13 and IL-5 (Th2 cytokines). Figure **C **shows the involvement of Lck (420983; 1 μM) and PKC (AEB071; 1 μM) signal transduction on PMA/CD3-induced IFNγ production. Figure **D **shows the effect of Lck (A-420983; 1 μM)) and PKC (AEB071; 1 μM) signal transduction pathways on PMA/CD28-induced IL-13 production. Data are presented as results from 3 donors measured as biological duplicates. Significance of differences are presented as followed; * *p *< 0.05, **p < 0.01 using a one-way ANOVA with Dunnett's post hoc testing.

A third example is provided by genes clustering together with EGR1, which show an up regulation by all stimuli but are specifically regulated by AEB071 in all conditions and only by A420983 after CD3/CD28 stimulus. Although IL-2 and EGR1 show a similar regulation by the stimuli used, the profiles can clearly be discriminated by the effects of the various inhibitors on their expression. The list of genes that are shown in Figure 7 together with their annotation and the correlation score to the CCL, IL-2 and EGR1 profiles are shown in Additional file 1: Figure S1, Additional file 2: Table S2, Additional file 3: Table S3

This analysis shows that by applying multiple stimuli and selective compound treatments, pathways can be unraveled at high resolution.

### Translation of PMA/CD3 and PMA/CD28 stimulations; differential modulation of primary T cell cytokine responses in human donor blood

In order to assess whether the stimulation profiles and signal transduction profiles identified in Jurkat T-cells were also relevant in a primary human setting, the stimulation protocols were adapted and a primary assay was established using healthy human donor whole blood. The effect of differential stimulation was evaluated using IFNγ and IL-17 as Th1- and Th17-associated read-outs respectively and both IL-5 and IL-13 as Th2-associated read-outs. Stimulation of human whole blood cells with CD3/CD28 was unsuccessful: no cytokine release was detected. However, PMA/CD3 stimulation of human blood cells, resulted in a high production of IFNγ (Figure 7A). Interestingly, INFγ production levels were significantly lower after PMA/CD28 stimulation. A similar observation was seen when analyzing IL-17 production. Thus, higher production levels of both IFNγ and IL-17 were seen following PMA/CD3 stimulation when compared to PMA/CD28 stimulation. Furthermore, when analyzing Th2-associated IL-5 and IL-13 production, we found that PMA/CD28 stimulation was superior to PMA/CD3 stimulation in enhancing production of these cytokines (Figure 7B). Of note, CCL1 production could not be detected in this assay system (data not shown). In aggregate, the data suggests that PMA/CD28 stimulation favours Th2 responsiveness in this assay. Since PMA/CD28 signaling was shown to be independent of Lck, but mainly dependent on PKCθ, whereas PMA/CD3 signaling was both Lck and PKCθ dependent we evaluated the effect of both proximal kinases in this human whole blood assay and evaluated IFNγ and IL-13 production since these cytokines were most readily produced. Figure 7C shows that indeed PMA/CD3-induced IFNγ production is depending on both Lck and PKCθ signaling, whereas PMA/CD28-induced IL-13 production is Lck-independent and PKCθ dependent. These results clearly show that the differential stimulations identified in the Jurkat assay can be translated towards a primary human cellular assay and are depending on the same proximal signaling hubs. Furthermore, also in this setting it can be observed that PMA/CD3 stimulation diverges more towards a Th1-like phenotype, whereas PMA/CD28 stimulation skews more towards a Th2-like response.

### PMA/CD3 stimulation of purified human CD4+ T cells enhances Th1 activation, whereas PMA/CD28 potentiates Th2 activation

**Figure 8:**
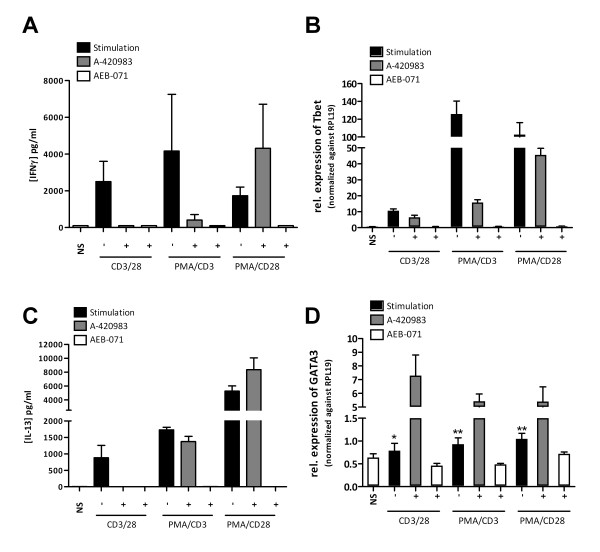
**PMA/CD3 and PMA/CD28 effects on purified primary human CD4+ T cells**. Purified CD4+ T cells were stimulated with anti-CD3/CD28, PMA/CD3 and PMA/CD28 and cultured for 24 hours in the presence or absence of pathway inhibitors for Lck (A420983; 1 μM) and PKC(AEB071; 1 μM). Culture supernatants were analyzed for IFNγ, production **(A)**, mRNA expression of the Th1 master transcription factor T-bet (**B)**, the production of the Th2 cytokine IL-13 **(C) **and expression of the Th2 master transcription factor GATA3 **(D)**. Message RNA expression was normalized against ribosomal house hold gene RPL19. Data are presented of three independent experiments using purified human CD4+ T cells from three different healthy donors. Data are presented as mean + SD. Significance of differences are indicated by ** *p *< 0.01, * *p *< 0.05, using a mann-withney U-test.

Using purified human CD4+ T cells we validated the effects observed on Jurkat T cells and in a primary human whole blood assay setting. Of all stimuli used PMA/CD3 appeared to be the most powerful stimulus able to induce IFNγ production. Also in this setting inhibition of either Lck using A-420983, or PKC using AEB071 completely inhibited PMA/CD3 induced IFNγ production. CD3/CD28-mediated stimulation, which can be successfully applied in this assay format induced IFNγ production, which was dependent on both Lck and PKC mediated signal transduction pathways. Of interest and comparable to the effects observed on CCL1 production by Jurkat T cells, Lck inhibition under PMA/CD28 stimulation did not inhibit IFNγ production and even appears to slightly enhance IFNγ production (Figure 8A). The observed effect on IFNγ production of the different stimuli is in line with the effects observed on the induction of the Th1 master transcription factor Tbet (Figure 8B). Both inhibition of Lck and PKC reduced CD3/28 and PMA/CD3 mediated induction of Tbet, whereas Lck inhibition did not affect PMA/CD28-induced expression of Tbet.

PMA/CD28 was the most profound inducer of IL-13 in CD4+ cells (Figure 8B). Interestingly IL-13 production under al stimulatory conditions used is dependent on PKC, whereas Lck inhibition does not affect IL-13 production under PMA/CD3 or PMA/CD28 culture conditions. Under all culture conditions inhibition of PKC reduced IL-13, which was paralleled with reduced GATA3 expression (Figure 8D), whereas inhibition of Lck appeared to promote Th2 dvelopment under all stimuli used, which was reflected by enhanced expression of GATA3.

## Conclusions

In this study we systematically explored pathways involved in T cell activation by molecular profiling. We showed that TCR (both CD3/28 and PMA/CD3) driven stimulation profiles are truly distinct from co-stimulatory profiles mediated via PMA/CD28. Secondly, using selective inhibitors and siRNA we found that the proximal kinase Lck is involved in CD3 and not PMA/CD28 activation, whereas PKCθ appears to be a crucial central signaling kinase in both TCR and PMA/CD28 (co)-stimulatory activation of T cells. Finally, stimulations involving TCR/CD3 appear to preferentially induce a Th1-like fingerprint, whereas lack of TCR/CD3 signaling in the presence of PMA/CD28 stimulation diverts T cells towards a Th2-like state.

It has been suggested that the strength of TCR-signaling can regulate the fate determination of naive T cells; high-potency signals skew towards Th1 differentiation, whereas low potency signals promote Th2 differentiation [6,22]. Although TCR and co-stimulatory pathways have been the focus of many studies in the previous decades, the direct contribution of TCR stimulation vs. co-stimulatory signals towards Th differentiation is not fully understood. By stimulating T cells with PMA/CD3 and PMA/CD28 we dissected signaling pathways and explored the activation profiles. CD3-mediated signaling rapidly increased intracellular Ca^2+^, a second messenger to activate many enzymes including Calcineurin, which resulted in an increased nuclear translocation of NFATc1. Interestingly, PMA/CD28 stimulation did not result in a Ca^2+^-mediated response (and was therefore marked a calcium-independent stimulation) but enhanced many of the co-stimulatory mediators including MAPK/AP1 and NFκB signal transduction. These results are in line with earlier studies that showed differential effects of cyclosporine A (CsA) and dexamethasone on CD3 vs CD28-mediated signaling, which revealed that PMA/CD28 stimulation was insensitive towards CsA-mediated Calcineurin inhibition in contrast to PMA/CD3 stimulation [20,23].

Gene expression induced by combinations of stimulatory signals revealed pathway-specific biomarkers or fingerprints. PMA/CD3-induced gene profiles included IL-2, IFNγ, XCL1, granzyme B, and FASL, which have been associated with a Th1 type of response [24,25]. Also, sustained NFAT signaling, which is also induced via PMA/CD3 stimulation, has been shown to promote Th1-like gene transcripts, including IFNγ, FasL and P-selectin glycoprotein ligand 1 [26]. Our results are further substantiated by the finding that T-bet (TBX21), the Th1 master transcription factor [27], and RUNX3, which together with Tbet are crucial for inducing IFNγ and repressing IL-4 [28], were highly expressed under PMA/CD3-stimulatory conditions.

PMA/CD28 stimulation does not induce a Ca^2+ ^flux nor does it increase nuclear translocation of NFAT. However it provides the cell with a high level of co-stimulatory signaling, and induces a completely distinct genomic fingerprint compared to PMA/CD3 stimulation. Following PMA/CD28 stimulation, Jurkat T cells highly expressed CCL1/I309, a chemokine which is highly expressed during a Th2-eosinophil response in allergic airway diseases [29,30]. Lymphotoxin (LT), a cytokine which is associated with a Th2-type of response controlling IgE production [31], was also highly expressed under PMA/CD28 stimulation. In conjunction with this finding, the master transcription factors for Th2, GATA3 [32] and the Retinoid X Receptor (RXR) [33], were induced under the PMA/CD28 stimulatory condition. Notably, Th2-associated cytokines like IL-4, IL-5 and IL-13 were not induced in Jurkat T cells after PMA/CD28-stimulation, this in contrast with PMA/CD28 stimulation of human whole blood and purified CD4+ T cells, which could be due to the developmental blockage of Jurkat T cells. Additional file 6: Figure 2 shows a schematic overview summarizing the involvement of the signaling pathways and genes induced under differential stimulation as observed in this study and highlights their relation towards T helper 1 and -2 development.

Our results are in line with the notion that high calcium levels drive Th1 and CTL responses and low calcium levels drive Th2 responses [7,34,35], which was further substantiated by our results using inhibitors for Lck and Cn, which modulate Calcium signaling in T cells. These inhibitors repressed Th1-associated genes under PMA/CD3-stimulation, but induced Th2 transcription factors GATA3 and RXRA, revealing a skewing of Th1 towards Th2 profiles. In contrast, PMA/CD28 stimulation in the presence of Lck and Cn inhibition, Th2-associated genes, e.g. CCL1 or IL-13 in CD4+ T cells, were not affected or even induced. The crucial role of Calcium and Lck in driving Th1 response is in line with the observation that knock down of Lck affects the virus-specific Th1/CTL response in mice and Lck deficiency increases Th2 associated cytokine production [36,37]. Interestingly, lack of Calcium signaling can give rise to an anergic T cell phenotype (reviewed in [38]). Therefore it would be of interest to further explore the role of Lck in calcium-dependent activation via PMA/CD3 on Th1/CTL responses and calcium-independent activation of T cells via PMA/CD28 on the induction of anergy in more detail.

CD28 signaling has been functionally linked with PKCθ induced activation of NFκB [39], which was also validated using PMA/CD28 as stimulus [40]. Previously it has been reported that CD28-costimulation induces GATA3 expression and Th2 differentiation via the activation of NFκB [41,42]. Additional studies in mice revealed that PKCθ is involved in mounting both Th2- and Th1-mediated lung inflammation, although Th2-mediated inflammation is more PKCθ-dependent [43]. Our studies show that inhibition of PKCθ can indeed inhibit a PMA/CD28 stimulation, which was reflected by the effect of PKCθ inhibition on the PMA/CD28-induced Th2-like gene expression profile. These observations are in line with the results from CD28 knock-out mice and inhibition of CD28 signaling using CTLA4Ig, showing that the CD28 co-stimulatory signaling is crucial for mounting a proper Th2 response. In contrast, Th1 and CTL responses were found to be less dependent on CD28 signaling [44,45]. Of interest, PKCθ inhibition in our hands, also affected PMA/CD3-induced Th1-like expression profiles. These results underline the duality of PKCθ in the integration of TCR and CD28-mediated signaling events which is evident from PKCθ KO mice experiments.

Finally, our results also show that this differential stimulation does not only occur in Jurkat T cells, but also plays a role in primary human T cells. These cells were found to secrete a Th1-like response (Tbet-IFNγ) via PMA/CD3 stimulation, whereas PMA/CD28 stimulation led to a Th2 activation profile (GATA3-IL-5/IL-13). In these cells inhibition of the Lck/Cn/NFAT pathway was only effective after PMA/CD3 stimulation whereas inhibition of PKCθ inhibited both PMA/CD3-induced IFNγ production and PMA/CD28-induced IL-13 production. These results illustrate that the findings in the Jurkat T cell line were successfully translated and relevant to a human primary cellular setting. Interestingly, PMA/CD3 stimulation also enhanced IL-17 production in the primary human whole blood assay and increased the expression of the IL-21 receptor, which is crucial for Th17 induction [46,47], in Jurkat T cells. These results suggest that additional signals, like IL-21 in conjunction with TGFβ and IL-6, might be necessary to differentiate from a Th1-like phenotype towards a Th17 phenotype, whereas the absence of TGFβ in the presence of high levels of IL-2 will favor Treg development or stabilization. Therefore further exploration of these differential stimulations in the presence of defined/different cytokine stimuli could further elucidate T helper cell differentiation and establish sub-set specific genome profiles. The findings described in this paper offer a robust platform for in vitro activation of T cells, in which observed responses can be easily translated form Jurkat T cells, towards purified CD4+ T cells and even human whole blood. This can be of interest for efficiency and selectivity profiling of kinase inhibitors or for pathway-specific biomarker identification for future drug development and clinical studies.

## Methods

### Compounds

Inhibitors selectively targeting defined pathways used in this study were A-420983 (1 μM; Lck inhibitor) [48], AEB-071 (10 μM; PKCθ inhibitor) [49] and Cyclosporin A (1 μM; Calcineurin inhibitor). Additionally, inhibitors of the MAPK pathway, SP600125 (10 μM; pan JNK), PD98059 (10 μM; MEK1/2), Org 48762-0 (10 μM; P38) [50] were used. All compounds were dissolved in 100% DMSO. Maximal and final concentration of DMSO used in the culture assays was 0.1% v/v.

### Cell culture

Jurkat E6.2.11 T cells were cultured in DMEM F12 medium (#041-94895 M, Gibco) supplemented with 10% FBS (#10099-141, Invitrogen) and 80 U/ml penicillin/80 μg/ml streptomycin (#15140-122 Gibco). Cells were cultured at concentrations between 1-2 × 10^5 ^cells/ml at 37°C/5%CO_2_. Cells were stimulated for 15 minutes up to 24 hours with anti-CD3 (1 μg/ml, OKT3), anti-CD28 (1 μg/ml, pericluster CD28 #M1456 Sanquin, the Netherlands), PMA (10 ng/ml, Sigma, USA) and ionomycin (1 μg/ml, Sigma, USA), or combinations thereof.

For gene expression profiling Jurkat T cells were seeded in T25 culture flasks at a concentration of 1 × 10^6 ^cells/ml (1 × 10^7 ^cells in total) and cultured overnight at 37°C/5%CO_2_, one day prior to stimulation. On the day of the experiment cells were preincubated with the compound of interest for 30 minutes, followed by a stimulation with either CD3/CD28, PMA/CD28 or PMA/CD3, at concentrations of 10 ng/ml PMA, 1 μg/ml CD3 and 1 μg/ml CD28. Jurkat T cells were cultured in the presence or absence of stimulation for one or eight hours in total, after which the cells were washed in ice cold PBS. Thereafter cell pellets were collected and snap frozen at -80°C. Cell pellets were stored until further processing.

### Isolation and quality check of mRNA

Total RNA was isolated from Jurkat T cells using the RNeasy mini extraction kit (Qiagen # 74106) according to the manufactures' protocol. RNA was dissolved and diluted in RNAse free water and the RNA concentration was determined via Nanodrop analysis. The quality of total RNA was evaluated by capillary electrophoresis using an Agilent 2100 Bioanalyzer (Agilent Technologies, Palo Alto, Calif.)

Double-stranded cDNA was synthesized from 1.5 μg total RNA using the One-Cycle Target Labeling Kit (Affymetrix Santa Clara, CA), and used as a template for the preparation of biotin-labeled cRNA using the GeneChip IVT Labeling Kit (Affymetrix Santa Clara, CA). Biotin-labeled cRNA was fragmented at 1 μg/μl following the manufacturer's protocol. After fragmentation, cRNA (10 μg) was hybridized at 45°C for 16-17 hours to the Human Genome U133A 2.0 Array or the Human Genome U133 Plus 2.0 Array (Affymetrix, Santa Clara, CA). Following hybridization, the arrays were washed, stained with phycoerythrin-streptavidin conjugate (Molecular Probes, Eugene, OR), and the signals were amplified by staining the array with biotin-labeled anti-streptavidin antibody (Vector Laboratories, Burlingame, CA) followed by phycoerythrin-streptavidin. The arrays were laser scanned with an GeneChip Scanner 3000 6 G (Affymetrix, Santa Clara, CA) according to the manufacturer's instructions. Data was saved as raw image file and quantified using GCOS (Affymetrix).

### Statistical analysis

The .CEL files were analyzed with the R http://www.r-project.org and the BioConductor software package http://www.bioconductor.org. Normalization was done using gcrma. Building of the experimental design and calculation of the ratios was done with the limma package. Regulated probe sets were selected on basis of the fold change and the adjusted p-value (Benjamini-Hochberg correction). Multivariate data analysis and clustering was done with standard methods in the R software package http://www.r-project.org. For the principal component analysis and hierarchical clustering, ratio data were used. The ratio data were calculated for each treatment to its corresponding control. For the treatment with the stimuli, the untreated cells were taken as a control. For the treatment with stimulus + compound combinations, the treatment with the stimulus alone was taken as a control.

Results were expressed as mean ± SEM. Significance of differences was determined using a one-way ANOVA followed by post-hoc testing as indicated.

Data sets can be found in GEO http://www.ncbi.nlm.nih.gov/gds/ under accession number GSE30678.

### FLIPR calcium flux assay

96-wells plates were coated with poly-L-lysine in PBS for 1 h at 37°C. Jurkat T cells were seeded at a concentration of 7 × 10^5 ^in culture medium and rested for 1 hour at 37°C/5%CO_2_. Thereafter cells were incubated for 1 hour in the dark with FLIPR calcium buffer, according to the manufacturers' protocol. Stimuli were added via the Flexstation384 and calcium release was monitored in time (Molecular devices, Sunnyvale, USA).

### Western blotting and nuclear translocation assay

Cells were washed in ice-cold PBS and pellets were lysed on ice in lysis buffer (Biosource, cat nr FNN0011, supplemented with 1 × protease inhibitor cocktail Roche cat no 11873580051, 1 mM PMSF Fluka cat no 93482, phosphatase inhibitor cocktail I, Sigma cat no P2850, phosphatase inhibitor cocktail II, Sigma cat no P5726) followed by an incubation for 30 min on ice. The lysates were stored at -80°C until further analysis.

Phosphorylation of proteins from stimulated Jurkat cells were evaluated via western blot analysis. Briefly, samples were run on a 4-12% NuPage gels (#NP321BOX, Invitrogen) for 35 min on 200 V in 1 × MES buffer (#NP0002, Invitrogen, USA) and subsequently transferred to a PVDF membrane (#162-0184, Biorad). The blots were blocked in PBS/0.05% Tween-20 with 1% skim milk (#232100, Difco) and 1% BSA. Blots were incubated O/N at 4°C in a roller bottle with the primary antibody diluted 1:1000 in block buffer, followed by incubation with a secondary detection antibody. Thereafter blots were incubated in ECL (#RPN2106V1 and RPN2106V2 Amersham Pharmacia) and hyperfilms (#RPN3103K, Amersham Pharmacia) were exposed and developed. For the detection the following antibodies were used: pLck/Src (Tyr^416 ^Cat N° 2101 L), Lck (Cat N° 2752), pZAP70 (Tyr^319 ^Cat N° 2701), pPKCθ (Thr^538^, Cat N° 9377), pMARCKS (Ser^152/156 ^Cat N° 4273) and ATF-2 (Thr^71 ^Cat N° 9221) (all cell signaling technology, Danvers, USA). pP38 (Thr^180^/Tyr^182 ^Cat N° 44-684 G), pERK (Thr^185^/Tyr^187 ^Cat N° 44-680 G), pJNK1/2 (Thr^183^/Tyr^185 ^Cat N° 44-682 G), JNK1 (Cat N° 44-690 G) were obtained from Invitrogen (Carlsbad, USA) and c-Jun (pSer^73^) was obtained from calbiochem (cat no 420114). For the analysis of nuclear translocation of the transcription factors NFAT, NFkBp65 and c-JUN, nuclear fractions of activated Jurkat T cells were isolated via hypotonic shock and levels of activated transcription factors in the nuclear lysates was tested in a TransAM transcription factor ELISA according to the manufacturers' protocol (Active Motif, Carlsbad, USA).

### Knock down of PKCθ and lck in Jurkat T cells

Jurkat T cells (2 × 10e7 cells/ml) were mock transfected or electroporated (250 V/975 μF) with siRNA targeting Lck (RefSeq accession-number NM-005356; sense 5'-GCACGCUGCUCAUCCGAAAdTdT) and PKCθ (RefSeq accession-number NM-006257; sense 5'-GCAGCAAUUUCGACAAAGAdTdT) in different concentrations of 500, 100 and 20 nM or scrambled control siRNA (Thermo scientific, Dharmacon Inc, Lafayette, USA). Electroporated cells were cultured in M505 supplemented with 10% FCS. Cells were stimulated 72 hours after electroporation and knock-down efficiency of the specified proteins was checked via western blot analysis. Culture supernatants of PMA/CD28 or PMA/CD3-stimulated cells were collected after 24 hours and production of respectively CCL1 and IL-2 was determined.

### Experiments with WBA

Peripheral whole blood was obtained by venipuncture from healthy adults (male/female) and was collected into lithium heparinised tubes. Blood was obtained from healthy volunteers and the time between puncture and processing was less than 1 hour. Blood was diluted 1:4 with RPMI 1640 (Life Technologies, cat no. 32404-014) supplemented with penicillin/streptomycin (GibcoBRL, cat 15140-122) and 2 mM L-glutamine (GibcoBRL, cat 25030-024) and distributed (200 μl/well) into 96-wells plates (Nunc, Cat 167008). Blood cultures were stimulated with soluble αCD3/PMA and soluble αCD28/PMA, or left unstimulated. Blood (200 μl/well) in RPMI 1640 medium was incubated with 25 μl compound (maximum of 0.1% v/v DMSO).

### Cytokine determination

Cytokines and chemokines IL-2, CCL1/I309 and XCL1, secreted into the supernatant of stimulated Jurkat T cells were determined via ELISA (R&D systems, USA). Cytokines IL-17, IFNγ, IL-13 and IL-5, produced by human CD4+ T-cells activated in whole blood, cultured in the presence or absence of compounds were determined in the culture supernatant, using a bead-based human cytokine multiplex kit (Bioplex-system; Bio-Rad, Veenendaal, The Netherlands) according to the manufacturer's instructions. Culture suppernatants were collected at day 1 of culture. Samples were analyzed using a Luminex-100 analyzer (Luminex, Austin, USA) with Bio-plex Manager Software 3.0 (Bio-Rad). Proteins were discriminated based on the fluorescent label of the bead and the PE levels were corrected for background levels of negative controls. The sensitivity of the cytokine assay was less than 5 pg/ml for all cytokines measured.

### cDNA synthesis and q-PCR

Primary human CD4+ T cells were isolated from buffy coats from three healthy donors using MACS negative CD4+ purification technology (Miltenyi biotech, Germany), yielding a overall 96% pure CD4+ T cell population. RNA from stimulated CD4+ T cells was isolated using an RNeasy minikit (QIAGEN Gmbh, Germany). RNA content of samples was analyzed using Nanodrop (Agilent technologies, Ca, USA) and purity was analyzed using the Agilent RNA 6000 nanokit protocol on the RNA nano labchip using the Agilent 2100 bioanalyzer (Agilent technologies, Waldborn, Germany). Three microgram of RNA was used for cDNA synthesis using random hexamer primer mix (invitrogen), 10 mM dNTP, M-MLV RT buffer and M-MLV Reverse transcriptase (Promega). RT reaction was performed at 42°C for 1 hour followed by a deactivation for 5 minutes at 90°C. cDNA of the Th1 master transcription factor Tbx21 (Tbet), Th2 transcription factor GATA3 or the control household gene RPL19 was applified using Power SYBR green mastermix (Applied Biosystems, Warrington, UK) and expression was monitored on the ABI prism 7900 HT sequence detection system (Applied Biosystems, Warrington, UK), Ct values were normalized for the expression of the RPL19 gene.

## Abbreviations

AP1: Activator protein 1; APC: Antigen presenting cell; ATF: Activating transcription factor; CCL1: CC chemokine ligand 1; Cn: Calcineurin; CsA: Cyclosporin A; CTL: Cytotoxic T cell; CTLA4: Cytotoxic lymphocyte antigen 4; ERK: Extracellular-signal-regulated kinase; FASL: TNFSF6 Fas ligand; GATA3: T cell specific transcription factor binding to DNA sequence GATA; IFNγ: Interferon gamma; IL: Interleukin; ITAM: Immuno-tyrosine based activation motif; Itk: IL-2-inducible T cell kinase; JNK: c-JUN N-terminal Kinase; Lck: p56 Lymphocyte-specific protein tyrosine kinase; MAPK: Mitogen activated protein kinase; MARCKS: Myristoylated alanine-rich C-kinase substrate; MHC: Major histocompatibility complex; NFAT: Nuclear factor of activated T cells; NFκB: Nuclear factor kappa B; OX40: CD134; P38: p38 Mitogen-activated protein kinase; PCA: Principle component analysis; PDK1: 3-phosphoinositide dependent protein kinase-1; PI3K: Phosphatydylinositol-3-kinase; PIP: Phosphatydylinositolphosphate; PLCγ: Phospholipase C gamma; PKB/AKT: Protein kinase B; PKCθ: Protein kinase C theta; PMA: Phorbol 12-myristate 13-acetate; RUNX3: Runt-related transcription factor 3; RXRA: Retinoid X Receptor alpha; T-bet: T-box transcription factor 21 (TBX21); TCR: T cell receptor; Th: T helper cell; TNF: Tumor necrosis factor; ZAP70: Zeta-chain-associated protein 70.

## Competing interests

The authors of this manuscript have no conflicts of interest to disclose as described by the Journal. The work performed here was funded by NV Organon, now part of MSD, Merck.

## Authors' contributions

Contribution: RLS, WA, SB, PV, XH, JPMK, IJ, AG and AMHB designed research; RLS, FW, MG, HK, and WA performed research; RLS, WF, WA,, XH, JPMK, IJ, SB, AMHB analyzed data; and RLS, WF, WA, SB, XH, JPMK, IJ, and AMHB, wrote the paper. All authors read and approved the final manuscript.

## Supplementary Material

Additional file 1 **Figure S1. PCA using the ratio data vs. the respective controls**. Cells were treated for 8 hours with the single stimuli CD28, CD3 and PMA (black squares) and with the stimulatory combinations PMA/CD28 (blue circles) CD3/CD28 (green triangles) and CD3_PMA (red diamonds). The corresponding treatments with the stimulus + inhibitor combination are represented by the same symbols. Eg. the red diamond with the AEB071 label represents the samplein treated with CD3 + PMA and AEB071. This graph shows that PMA/CD28 stimulation was mainly affected by AEB071, whereas PMA/CD3 stimulation was mainly modulated by CsA, A420983 and AEB071. The symbols around the origin represent the treatments with the MAPK inhibitors, which only had a marginal effect on the stimuli used (the labels have been omitted for clarity). The numbers denote the number of significantly regulated probe sets for these conditions compared to the respective controls. These numbers were very low for the conditions including MAPK inhibitors and CD28 stimulation alone and for A420983 and CsA after PMA/CD28 stimulation (centered around the origin of the graph) and have therefore been omitted for clarity. For details of regulated probe sets for all conditions and the overlap between these sets, see Additional file 2: Table S1. Repeating this analysis with different values for the fold change and p-value cut off yielded essentially the same results for the multivariate analysis. Click here for file

Additional file 2 **Table S1**. For each treatment, regulated probe sets were selected by comparison with the corresponding control. Probe sets were identified as being significantly regulated if adjusted p-value was < 10^-10 ^and fold change > 4. The numbers in each cell show the number of overlapping genes between two treatments. The cells highlighted in grey indicate the most informative numbers because in these comparisons, only one of the parameters is changed between two conditions. The other numbers are shown for the sake of completeness, but are less informative because the comparisons are between treatments in which two parameters at the same time have been changed. Click here for file

Additional file 3 **Files S1**. Each of these files contain 4 columns. The first is the Affymetrix probe set id, the second columns gives the Euclidean distance to the gene expression profile of interest (file 1: CCL1, file 2: IL-2, file 3: EGR1). The third and fourth columns show the corresponding gene symbol and description respectively. Click here for file

Additional file 4 **Files S2**. Each of these files contain 4 columns. The first is the Affymetrix probe set id, the second columns gives the Euclidean distance to the gene expression profile of interest (file 1: CCL1, file 2: IL-2, file 3: EGR1). The third and fourth columns show the corresponding gene symbol and description respectively. Click here for file

Additional file 5 **Files S3**. Each of these files contain 4 columns. The first is the Affymetrix probe set id, the second columns gives the Euclidean distance to the gene expression profile of interest (file 1: CCL1, file 2: IL-2, file 3: EGR1). The third and fourth columns show the corresponding gene symbol and description respectively. Click here for file

Additional file 6 **Figure S2**. A schematic presentation of signaling pathways and induced gene profiles via differential stimulation of T cells. This figure highlights the findings of this study indicating that TCR/CD3-induced Calcium signaling is necessary for efficient T helper 1 development, whereas absence of calcium signaling and sufficient activation of NFκB/AP1 lead to T helper 2 development (as indicated by green-red intensity plots). A selected list of genes is listed derived from the IL-2 and CCL1 gene profiles shown in Figure 7. Click here for file
