# Supplementary figures and images for: Molecular pathway profiling of T lymphocyte signal transduction pathways; Th1 and Th2 genomic fingerprints are defined by TCR and CD28-mediated signaling
Source: BMC Immunol. 2012 Mar 14;13:12. doi: 10.1186/1471-2172-13-12 (PMC3355027; doi:10.1186/1471-2172-13-12)

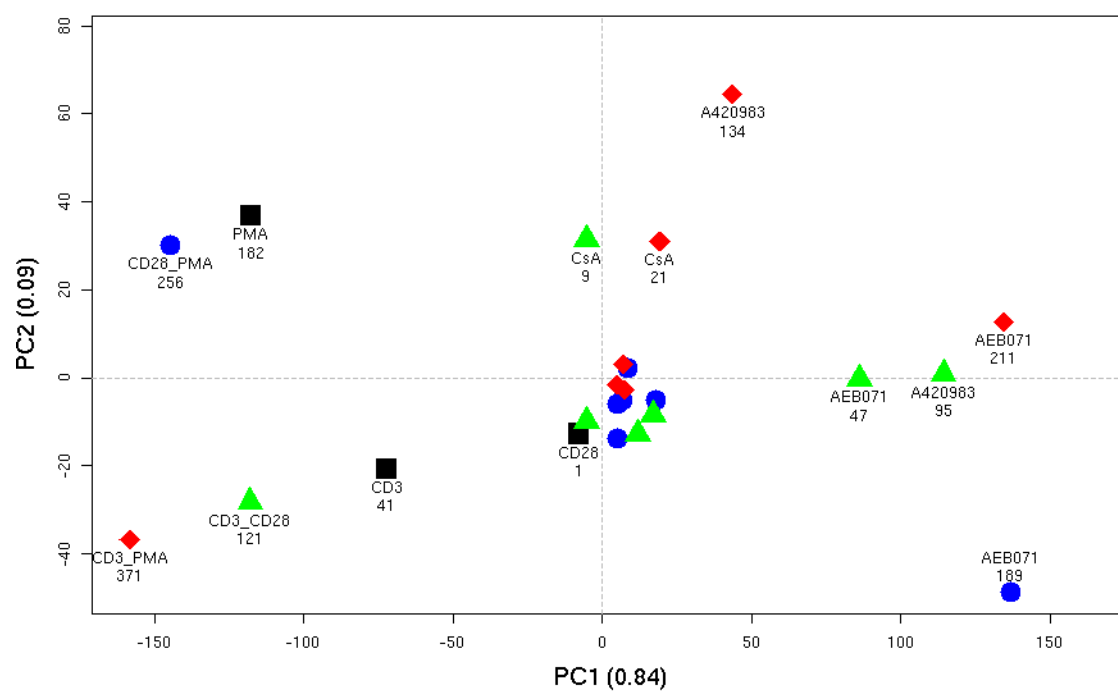

Supplement: Additional file 1 — Figure S1. PCA using the ratio data vs. the respective controls. Cells were treated for 8 hours with the single stimuli CD28, CD3 and PMA (black squares) and with the stimulatory combinations PMA/CD28 (blue circles) CD3/CD28 (green triangles) and CD3_PMA (red diamonds). The corresponding treatments with the stimulus + inhibitor combination are represented by the same symbols. Eg. the red diamond with the AEB071 label represents the samplein treated with CD3 + PMA and AEB071. This graph shows that PMA/CD28 stimulation was mainly affected by AEB071, whereas PMA/CD3 stimulation was mainly modulated by CsA, A420983 and AEB071. The symbols around the origin represent the treatments with the MAPK inhibitors, which only had a marginal effect on the stimuli used (the labels have been omitted for clarity). The numbers denote the number of significantly regulated probe sets for these conditions compared to the respective controls. These numbers were very low for the conditions including MAPK inhibitors and CD28 stimulation alone and for A420983 and CsA after PMA/CD28 stimulation (centered around the origin of the graph) and have therefore been omitted for clarity. For details of regulated probe sets for all conditions and the overlap between these sets, see Additional file 2: Table S1. Repeating this analysis with different values for the fold change and p-value cut off yielded essentially the same results for the multivariate analysis. [file 1471-2172-13-12-S1.PDF]

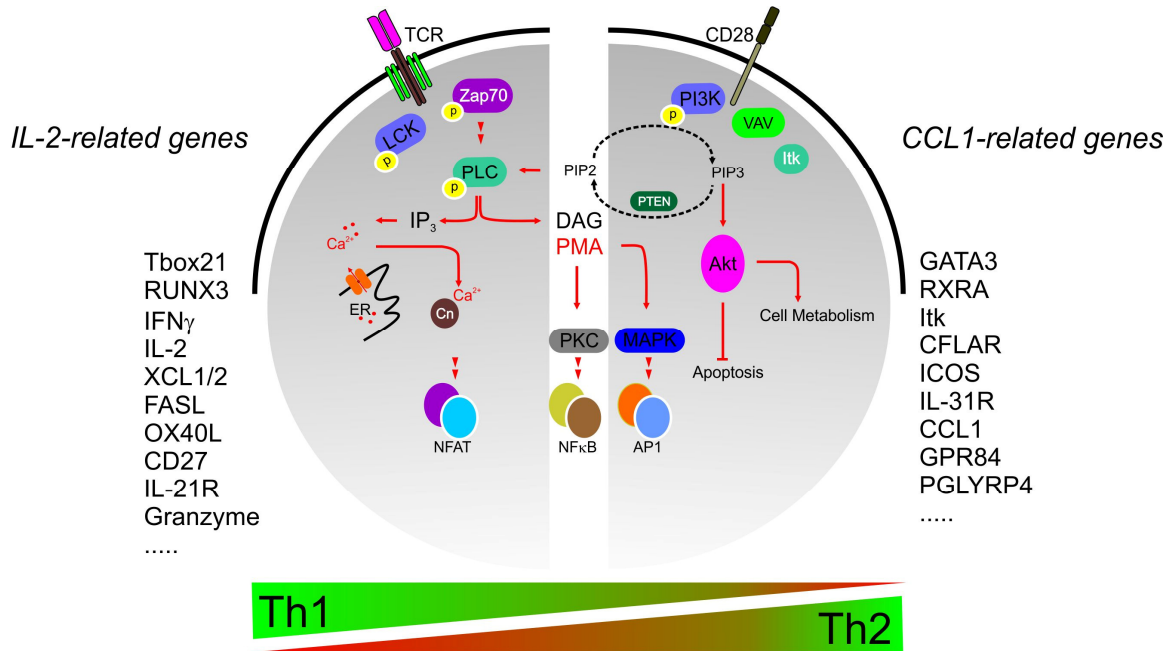

Supplement: Additional file 6 — Figure S2. A schematic presentation of signaling pathways and induced gene profiles via differential stimulation of T cells. This figure highlights the findings of this study indicating that TCR/CD3-induced Calcium signaling is necessary for efficient T helper 1 development, whereas absence of calcium signaling and sufficient activation of NFκB/AP1 lead to T helper 2 development (as indicated by green-red intensity plots). A selected list of genes is listed derived from the IL-2 and CCL1 gene profiles shown in Figure 7. [file 1471-2172-13-12-S6.PDF]
