# Supplementary material for: Molecular pathway profiling of T lymphocyte signal transduction pathways; Th1 and Th2 genomic fingerprints are defined by TCR and CD28-mediated signaling
Source: BMC Immunol. 2012 Mar 14;13:12. doi: 10.1186/1471-2172-13-12 (PMC3355027; doi:10.1186/1471-2172-13-12)
Supplement: Additional file 2 — Table S1. For each treatment, regulated probe sets were selected by comparison with the corresponding control. Probe sets were identified as being significantly regulated if adjusted p-value was < 10-10 and fold change > 4. The numbers in each cell show the number of overlapping genes between two treatments. The cells highlighted in grey indicate the most informative numbers because in these comparisons, only one of the parameters is changed between two conditions. The other numbers are shown for the sake of completeness, but are less informative because the comparisons are between treatments in which two parameters at the same time have been changed. [file 1471-2172-13-12-S2.PDF]

Stimulus	Stimulus		CD28	CD3	PMA	CD28_PMA	CD3_CD28	CD3_PMA			CD28_PMA						CD3_CD28						CD3_PMA			
Treatment	Inhibitor	Nr of probe sets							A420983	CsA	AEB071	ORG48762_	SP60012	PD9805	A42098	CsA	AEB071	ORG48762_0	SP600125	PD98059	A420983	CsA	AEB071	ORG48762_0	SP600125	PD98059
CD28		0	0	0	0	0	0	0	0	0	0	0	0	0	0	0	0	0	0	0	0	0	0	0	0	0
CD3		41	0	41	31	32	34	34	1	0	27	1	0	1	29	1	16	0	0	0	5	0	24	0	0	0
PMA		182	0	31	182	152	76	118	1	0	117	1	0	1	68	7	36	0	0	0	25	0	74	0	0	0
CD28_PMA		256	0	32	152	256	91	163	1	0	178	1	0	1	74	7	39	0	0	0	24	0	107	0	0	0
CD3_CD28		121	0	34	76	91	121	115	1	0	76	1	0	1	73	3	41	0	0	0	19	0	81	0	0	0
CD3_PMA		371	0	34	118	163	115	371	1	0	125	1	0	1	83	6	42	0	0	0	59	5	166	1	2	1
CD28_PMA	A420983	1	0	1	1	1	1	1	1	0	1	1	0	1	1	0	1	0	0	0	1	0	1	0	0	0
CD28_PMA	CsA	1	0	0	0	0	0	0	0	1	0	0	0	0	0	1	0	0	1	0	1	1	0	0	0	0
CD28_PMA	AEB071	189	0	27	117	178	76	125	1	0	189	1	0	1	64	5	38	0	0	0	21	0	103	0	0	0
CD28_PMA	ORG48762_	1	0	1	1	1	1	1	1	0	1	1	0	1	1	0	1	0	0	0	1	0	1	0	0	0
CD28_PMA	SP600125	0	0	0	0	0	0	0	0	0	0	0	0	0	0	0	0	0	0	0	0	0	0	0	0	0
CD28_PMA	PD98059	1	0	1	1	1	1	1	1	0	1	1	0	1	1	0	1	0	0	0	1	0	1	0	0	0
CD3_CD28	A420983	95	0	29	68	74	73	83	1	0	64	1	0	1	95	3	40	0	0	0	15	0	67	0	0	0
CD3_CD28	CsA	9	0	1	7	7	3	6	0	1	5	0	0	0	3	9	3	0	1	0	3	1	3	0	0	0
CD3_CD28	AEB071	47	0	16	36	39	41	42	1	0	38	1	0	1	40	3	47	0	0	0	8	0	45	0	0	0
CD3_CD28	ORG48762_	0	0	0	0	0	0	0	0	0	0	0	0	0	0	0	0	0	0	0	0	0	0	0	0	0
CD3_CD28	SP600125	3	0	0	0	0	0	0	0	1	0	0	0	0	0	1	0	0	3	0	1	1	0	0	0	0
CD3_CD28	PD98059	0	0	0	0	0	0	0	0	0	0	0	0	0	0	0	0	0	0	0	0	0	0	0	0	0
CD3_PMA	A420983	134	0	5	25	24	19	59	1	1	21	1	0	1	15	3	8	0	1	0	134	9	55	1	2	1
CD3_PMA	CsA	21	0	0	0	0	0	5	0	1	0	0	0	0	0	1	0	0	1	0	9	21	5	1	1	1
CD3_PMA	AEB071	211	0	24	74	107	81	166	1	0	103	1	0	1	67	3	45	0	0	0	55	5	211	1	2	1
CD3_PMA	ORG48762_	1	0	0	0	0	0	1	0	0	0	0	0	0	0	0	0	0	0	0	1	1	1	1	1	1
CD3_PMA	SP600125	2	0	0	0	0	0	2	0	0	0	0	0	0	0	0	0	0	0	0	2	1	2	1	2	1
CD3_PMA	PD98059	2	0	0	0	0	0	1	0	0	0	0	0	0	0	0	0	0	0	0	1	1	1	1	1	2

Supplemental table 1
